# Empyema thoracic in a neonate co-infected with SARS-CoV-2 and staphylococcus arouse successfully treated with fibrinolysis: a brief report

**DOI:** 10.1186/s12887-023-04375-6

**Published:** 2023-11-03

**Authors:** Fatemeh Sabzevari, Reza Sinaei, Aazam Gholami, Farzad Tahmasbi

**Affiliations:** 1 Department of Pediatrics, Afzalipour Hospital, Kerman University of Medical Sciences, Kerman, Iran; 2 Department of Pediatrics, Kerman University of Medical Sciences, Kerman, Iran; 3 Endocrinology and Metabolism Research Center, Institute of Basic and Clinical Physiology Sciences, Kerman University of Medical Sciences, Kerman, Iran; 4 Clinical Research Development Unit, Afzalipour Hospital, Kerman University of Medical Sciences, Kerman, Iran

**Keywords:** COVID-19, SARS-CoV-2, Empyema, Neonate, Fibrinolysis

## Abstract

**Background:**

Empyema as a rare cause of respiratory distress in neonatal period has not yet been reported after Corona virus disease 2019 (COVID-19) and even rarely in the context of bacterial infections is age group.

**Case Presentation:**

The first neonate of quadruplets, resulting from Cesarean-Section of a 30-year-old mother without a history of previous illness, born at 34 weeks of gestation with a birth weight of 1600 gram. All four newborns were hospitalized; while the first one underwent nasal continuous positive airway pressure (N-CPAP) with a positive end-expiratory pressure of 6 and fractional inspired oxygen of 0.6, due to respiratory distress, noting a respiratory score of five out of 12.She was resuscitated one hour later due to bradycardia and underwent ventilator support. She received a single dose of pulmonary surfactant, intravenous paracetamol, and phenobarbital due to respiratory distress syndrome, Pectus Ductus Arteriosus, and unilateral clonic movements, respectively. In the lack of positive blood culture, she extubated two days later and breast-feeding was started. On day eight, she underwent high flow nasal cannula. On day 12, she suddenly developed respiratory distress, mottling, and abdominal distension, leading to N-CPAP and re-evaluation. The nasopharyngeal sampling for severe acute respiratory syndrome coronavirus-2 (SARS-CoV-2) real time Polymerase chain reaction and the blood culture for staphylococcus aurous became positive. A large volume pleural effusion including septa and loculation in right hemi-thorax on chest computerized tomography scan and ultrasound was seen, necessitating to thoracotomy, which was not possible due to her general condition. Remdesivir was started and antibiotics changed to a more broad-spectrum coverage. Chest tube was inserted and Alteplase was injected for three consecutive days with beneficial effects. She had no problem for six-month follow up.

**Conclusion:**

This was a case of empyema thoracic in the context of SARS-CoV-2 and Staphylococcus arouses co-infection. In our experience, it can be treated by chest drainage and fibrinolysis in neonatal period.

## Introduction

Corona virus disease 2019 (COVID-19) clinical presentations and complications are more prominent in adults than in pediatric population [1]. However, several extra-pulmonary complications are still being reported in children [2, 3].

Studies exploring the impacts of COVID-19 on newborns are still limited. Although several studies considered the vertical transmission unlikely, some indicated the possibility of vertical transfer. Presently, few respiratory involved newborns have been reported during COVID-19 pandemic and the majority of them have mild to moderate symptoms [4]. However, pleural effusion is a rare presentation of COVID-19. Empyema as a purulent pleural fluid usually arises from Para-pneumonic effusion from bacterial infection, resulting in pleural inflammation [5]. The impact of both superadded and opportunistic infections is compounded even more by the recent variants, developing empyema in both pediatric and adult affected groups with COVID-19 [6, 7]. In addition, culture negative empyema in a 75-year-old patient after COVID-19 resolution has been raised the hypothesis that delayed inflammation especially in the context of immune defect might be responsible [8]. Considering the entity of the immunity in the newborns that may limit both localization of infection to the pleural space and capacity of the pleura to induce enough exudates, it is rarely seen in neonatal period, even following severe pneumonia [9].

There is still no accepted protocol in the management of neonatal empyema thoracic. The treatment usually consists of pleural fluid drainage, chest tube insertion, and prompt initiation of antibiotics, but little is known regarding the surgical approach and fibrinolysis in this period [10].

Here, we present a SARS-CoV-2 and Staphylococcus aurous co-infected newborn that developed to empyema thoracic. To our knowledge, using the fibrinolysis is uniqueness and a less experienced procedure in neonatal period.

## Case presentation

A girl neonate as the first baby of quadruplets with birth weight of 1600 gram born by Cesarean Section (CS) in Afzalipour Hospital, located in Kerman, southeast of Iran. She born from a 30-year-old gravid-I mother with gestational age of 34 weeks, while she was in transverse presentation.

The mother had no history of previous illness or medications. She had received betamethasone at 28 weeks of gestation and eventually underwent CS due to shortness of breath and multiple pregnancies. She had no history of fever, constitutional symptoms and clear contact with infections patients.

All four neonates were hospitalized, while the first one Apgar score was eight and nine out of ten in first and five minutes, respectively. She had respiratory distress at the time of birth, presenting with tachypnea (Respiratory rate of 72 breaths per minute), subcostal retraction, reduced bilateral lung sounds, mild hypotonia, and grunting. Her temperature was 36.5 ˚C, and the pulse rate was 148 beat/min, noting a holo-systolic murmur based on Levine classification, which was III out of IV in intensity and was auscultated especially in the upper part of left sternal border. She underwent nasal continuous positive airway pressure (N-CPAP) with positive end-expiratory pressure (PEEP) of 6 and fractional inspired oxygen (FIO2) of 0.6 due to respiratory distress, noting a respiratory score of five out of 12 based on Down’s score. She underwent sepsis work up and Ampicillin (50 mg/kg q 8 h) and Gentamycin (2.5 mg/kg/dose q 12 h) were prescribed, empirically. One hour later, given the progressive nature of symptoms, developing to bradycardia and decreased oxygenation, the patient was resuscitated step by step according to the neonatal resuscitation program and resuscitation guidelines of American Academy of Pediatrics with chest compression and Positive Pressure Ventilation. Her vital signs returned after two minutes. She was finally underwent mechanical ventilation of pressure controlled mandatory ventilation with RR, FIO2, Inspiratory/Expiratory (I/E) ratio, PEEP, and Pressure Control(PC) of 40 breaths per minute, 0.5, 1/2, 6, and 8, respectively.

Considering the chest radiograph and clinical condition consistent with the diagnosis of respiratory distress syndrome (RDS), one dose of respiratory surfactant (Curosurf 80 mg/mL; 200 mg/kg) was prescribed.

The patient’s clinical symptoms including bounding pulse and holo-systolic murmur were consistent with the diagnosis of pectus ductus arteriosus (PDA). The echocardiography confirmed the diagnosis of PDA, and also showed a small atrial septal defect (ASD).According to cardiologic consultation intravenous paracetamol (Apotel) was started at a dose of 15 mg/kg every 6 h for three days with beneficial effect. The patient experienced two episodes of clonic seizure for near 30 s with tachycardia and decreation in O2 saturation. According to neurological consult, Phenobarbital was started with a loading dose of 20 mg/kg and a maintenance dose of 5 mg/kg/day twice a day and continued for two months. Electroencephalogram was not performed at beginning and was postponed to an outpatient examination.

Additional chest radiographs showed good lung aeration. She was extubated 48 h later without need for second dose of surfactant. She underwent nasal intermittent positive pressure ventilation (NIPPV) with a RR of 20 breaths per minute, FIO2 of 1.5/7, I/E ratio of 0.4, PEEP of 6, and PC of 9. The breast feeding was started with a gradually increasing pattern. At this time the blood and urine cultures were negative. Other laboratory investigations were included in Table 1.

**Table 1:** Laboratory investigations of patient

Laboratory parameter	Normal values	Day 1	Day 14 ± 2	At the last days of admission
White Blood Cell	4.5–10.0 $\times$ 10^3^	9.4	27.8	15
Lymphocyte %	Dominant	79.5	5.6	70.1
Hemoglobin	12.0–16 g/dL	19.4	15.5	11.6
MCV	110 ± 15 fL	98	95	91
Platelet	150–450 × 10^9^/UL	246	568	685
C-reactive Protein	Less than 5 mg/Dl	5.1	21.9	7
PH	7.35–7.45	7.4	7.35	7.3
HCO3	22–28 meq/L	22	16	21.6
PCO2	35–45 mmHG	33	30	48
Bili-Total	Less than 2.0 mg/dL	3.5	11	2.5
Bili-Direct	Less than 0.5 mg/dL	0.4	0.9	0.4
BUN	15–45 mg/dL	22	18	21
Creatinine	0.5–0.9 mg/dL	0.9	0.3	0.4
LDH	160–450 U/L	NA^1^	330	NA
Pleural Fluid Analysis		NA	Volume: 20 ml Gross: Turbid, purulent WBC count: 16,000/mcL Neutrophil: 89% LDH: 6500 U/L Protein: 5.6 g/dl Glucose: 20 mg/dl PH: 7.1 Gram stain: gram positive cocci	
Blood Culture	+/- (type)	Negative	Staphylococcus aureus	Negative
CSF Culture	+/- (type)	NA	Negative	NA
Urine Culture	+/- (type)	Negative	Negative	NA
Pleural Fluid Culture	+/- (type)	NA	Negative	Negative

^1^: not assessed

**Fig. 1:**
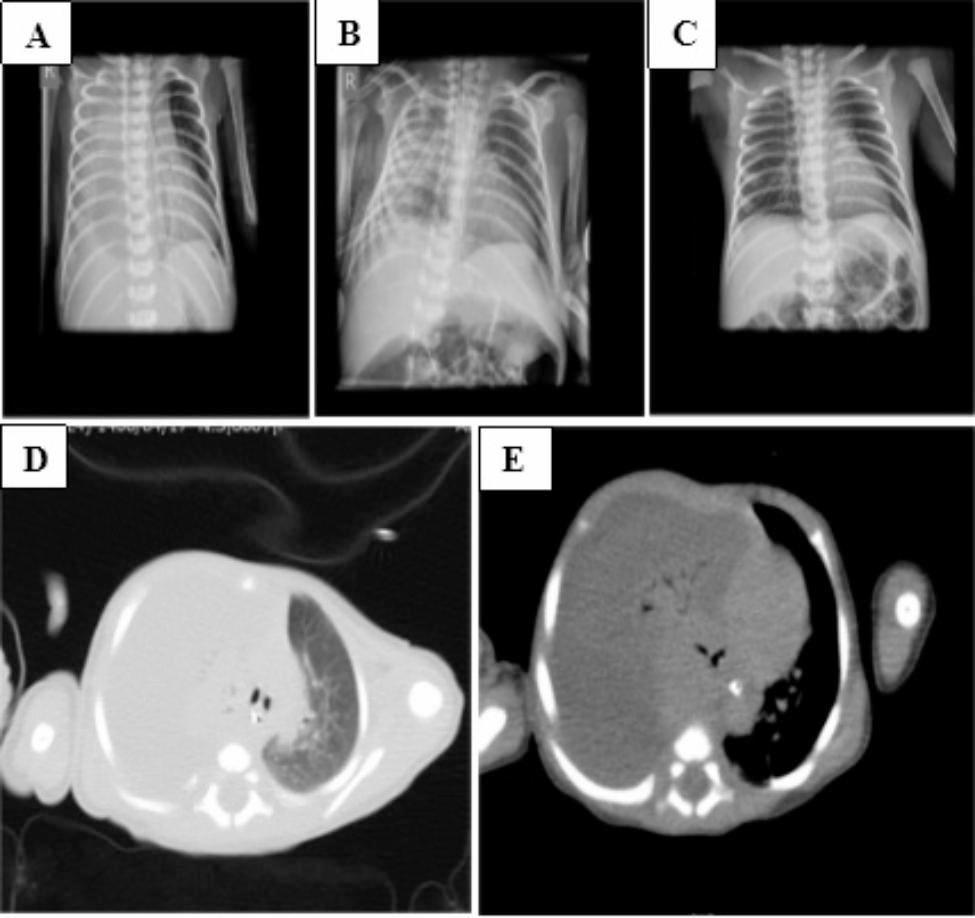
Chest radiographs of patient before (A) and after (B) chest tube insertion. Chest radiograph at the end of treatment (C). Chest CT-Scan showing a large volume of empyema on the right side (D-E).

On day eight, she underwent high-flow nasal cannula (HFNC) with FIO2 of 0.4 and a flow of 3 lit/min. On day 12, she suddenly developed respiratory distress, mottling, and abdominal distension, leading to N-CPAP and a systematic re-evaluation. The nasopharyngeal sampling for SARS-CoV-2 Polymerase chain reaction (RT-PCR) was performed with positive result. Moreover, the staphylococcus aureus grew in blood culture. The brain ultrasound and echocardiogram had no abnormality compatible with this condition. Chest radiograph was performed and right side costo-pherenic angle was blunt. A chest computerized tomography (CT) scan was done, showing whiteout right hemi-thorax due to large volume PE which causing right lung collapse and mediastinal shift to left side (Fig. 1). A large volume of fluid including debris, septa, and loculation suggestive of empyema thoracics was seen in chest ultrasound, necessitating to thoracotomy, which was not possible due to the patients’ poor general condition. The patient was still on N-CPAP with a PEEP of 7 and FIO2 of 0.4, while her arterial blood gas was in normal range. Remdesivir (5 mg/kg/day in the first day and 2.5 mg/kg/day for the last four days) and broad-spectrum antibiotics of Meropenem (20 mg/kg/dose; three doses for 21 days) and Vancomycin (10 mg/kg/dose; twice a day for 21 days) were prescribed according to the consultation with an infectious subspecialist. The chest tube was inserted through a minimal cut into the right pleural space and tissue plasminogen activator of Alteplase with a dose of 0.1 cc/kg (diluted with 1 cc/kg of 0.9% saline) injected into the pleural space. The chest tube was clamped for one hour and then opened. Alteplase was injected for three consecutive days with beneficial effects in both clinical and radiological investigations. The chest ultrasound after these days revealed diminished pleural fluid containing less debris and septa. The chest tube was extracted after 16 days and antibiotics were discontinued after 21 days.

Finally, the patient was discharged after 40 days of admission in a good general condition with negative blood culture and SARS-CoV-2 RT-PCR tests. Seven days later, the chest radiograph and ultrasound were performed and both were normal. EEG was normal at this time and phenobarbital discontinued after two months. The patient had no problem in her physical examination including her growth and development during one-year follow up. Other investigations including audiometry, thyroid function tests, and ophthalmic examination for premature retinopathy showed no abnormality.

## Discussion

Pleural empyema is a rare condition in neonatal period and is accompanied with high fatality rate [9–11]. There are still no therapeutic protocol in the management of this condition in neonates [12, 13].

The pathogens isolated from affected children are Hemophilus Influenza, Staphylococcus aureus, Streptococcus pneumoniae, bacteroides species, and other anaerobes [14]. Viral and mycoplasma pneumoniae have been implicated up to 20% of cases with para-pneumonic effusion [6].

Little is known regarding the para-pneumonic effusion and empyema happening in the context of COVID-19. There are few cases of childhood empyema following SARS-CoV-2 infection described in medical literature, with no report in neonatal period [7, 10]. However, both superinfection and hyper-inflammatory states have been found culprit in this regard [15]. Our patient had infection with S. aureus, which is one of the most common acquired bacterial pathogens resulting to pleural empyema. In addition, she had a SARS-CoV-2 positive testing. To our knowledge, this is the first newborn infected with SARS-CoV-2 and staphylococcus aurous resulted to empyema thoracic.

In the lack of a definite treatment strategy in this age group, the key for successful management lies in two basic principles, an effective antibiotic therapy and re-expansioning of the lung [14]. However, the management of complicated effusion by conventional first-line treatment with chest tube insertion and antibiotics may fail because of thick viscous entity of fluid containing septa and loculation, just like our patient. Intra-pleural fibrinolysis and video assisted thoracoscopic surgery (VATS) can be used, although the use of fibrinolytic in neonates has not been studies extensively [12, 16–20]. All case reports in neonatal population that described successful treatment with fibrinolysis are in term neonates, except Xerra and colleagues that described the beneficial effects of this therapeutic strategy in a premature neonate [17], just like of our patient.

In contrast, VATS can be an effective and safe modality and therapeutic option for neonatal empyema [14].

In contrast to our experience, a premature newborn that reported by Lohmeir and their colleagues successfully responded to conventional conservative treatment [13]. Our patient was resistant to first-line strategies, where pharmacological therapies and chest drainage were not sufficient to eradicate the infection. Since she could not tolerate surgical procedure due to her general condition, the fibrinolysis was performed as alternative strategy. Comparing the use of fibrinolysis to VATS in the treatment of empyema, studies in found no significant difference in the length of hospital stay and failure rate, but not cost, with fibrinolysis modality costing less [21, 22].

## Conclusion

Empyema as a Para-pneumonic complicated entity has not been reported yet following COVID-19 in the neonatal period. Here, we presented a SARS-CoV-2 and Staphylococcus aurous co-infected newborn that developed to empyema thoracic. To our knowledge, this is the first case of exclusive and successfully treated of empyema with fibrinolysis in a premature infant.

Although, chest drainage and antibiotics might represent an acceptable option in therapeutic strategies, complicated cases with thick and loculated fluid may need to other modalities including surgery or fibrinolytic.

## Data Availability

The datasets used and/or analyzed during the current study are available from the corresponding author on reasonable request.

## Abbreviations

COVID-19: Coronavirus disease 2019
CS: Cesarean-Section
N-CPAP: nasal continuous positive airway pressure
PEEP: positive end-expiratory pressure
FIO2: fractional inspired oxygen
RDS: respiratory distress syndrome
PDA: Pectus Ductus Arteriosus
HFNC: high flow nasal cannula
SARS-CoV-2: severe acute respiratory syndrome coronavirus-2
RT-PCR: real time Polymerase chain reaction
CT: chest tomography
ASD: small atrial septal defect
